# Thermal and Geometric Error Compensation Approach for an Optical Linear Encoder

**DOI:** 10.3390/s21020360

**Published:** 2021-01-07

**Authors:** Donatas Gurauskis, Artūras Kilikevičius, Albinas Kasparaitis

**Affiliations:** 1 Department of Mechanical and Material Engineering, Vilnius Gediminas Technical University, J. Basanavičiaus g. 28, 03224 Vilnius, Lithuania; albinas.kasparaitis@vgtu.lt; 2 Institute of Mechanical Science, Vilnius Gediminas Technical University, J. Basanavičiaus g. 28, 03224 Vilnius, Lithuania; arturas.kilikevicius@vgtu.lt

**Keywords:** measuring scale, thermoelastic deformation, coefficient of thermal expansion

## Abstract

Linear displacement measuring systems, like optical encoders, are widely used in various precise positioning applications to form a full closed-loop control system. Thus, the performance of the machine and the quality of its technological process are highly dependent on the accuracy of the linear encoder used. Thermoelastic deformation caused by a various thermal sources and the changing ambient temperature are important factors that introduce errors in an encoder reading. This work presents an experimental realization of the real-time geometric and thermal error compensation of the optical linear encoder. The implemented compensation model is based on the approximation of the tested encoder error by a simple parametric function and calculation of a linear nature error component according to an ambient temperature variation. The calculation of a two-dimensional compensation function and the real-time correction of the investigated linear encoder position readings are realized by using a field programmable gate array (FPGA) computing platform. The results of the performed experimental research verified that the final positioning error could be reduced up to 98%.

## 1. Introduction

The vast majority of industrial and scientific applications use optical encoders for a position measurement and closed-loop position control, for example, machine tools [1,2,3,4,5], tracking systems [6,7,8], industrial robots [9,10,11,12,13], positioning stages [14,15,16,17], and so on. All these technological machines work under various environment conditions such as temperature, humidity, mechanical vibration, etc. In turn, these effects inevitably generate a corresponding error. According to Ramesh et al. [18,19], the thermal factors account for 40–70% of the total dimensional and shape errors in machine tools. Much scientific research has been performed to analyze, model, and compensate the influence of thermal positioning error in manually or computer-numerical-control (CNC) machines [20,21,22,23,24,25,26,27,28]. The majority of this research analyzes only the machine tool structure, considering that the used measurement system is not the source of the error by itself [29].

Working environment adversely affects the accuracy of the integrated encoder. Lopez et al. investigate optical encoder errors under vibration at different mounting conditions [30] and optical scanning principles [31]. They also present a methodology for a vibration error compensation [32]. Performance of the encoder could be improved by correcting its output signals in real-time. This could be done by using look-up tables, digital filtering, or other techniques [33,34,35,36,37]. These methods help to reduce a high frequency sub-divisional encoder error that repeats at each period of a scale grating. Temperature changes introduce strains that change the width of a grating period. Therefore, the thermal errors could not be compensated by using these techniques.

The thermal behavior of the encoder is also a very important factor. One way to deal with a changing temperature impact is by using low thermal coefficient (CTE) materials. Glass ceramics like ZERODUR $\left(\mathrm{CTE}=0\pm 0.1\;µm/m^{\circ }C\right)$ or ROBAX $\left(\mathrm{CTE}=\sim 0\;µm/m^{\circ }C\right)$ are used for a measuring scale manufacturing. Another way is trying to match the thermal coefficients between used linear encoder, machine tool support, and a workpiece material. In this case, the change in a workpiece size has the same value as the expanded or contracted encoder, so the thermoelastic error is practically eliminated. A well-defined and reproducible thermal behavior of the encoder must be ensured. Unfortunately, it is quite challenging task to do. For example, an enclosed type linear encoder consists of an aluminum extrusion and a measuring scale, usually made from glass or stainless steel. The scale is attached to extrusion by adhesive or a double-sided adhesive tape. Such an assembly demonstrates a complex thermal behavior because of the combination of different CTE materials. Alejandre et al. [38] present the method to determine the real thermal coefficient of a linear encoder. In this research, they investigate an enclosed-type linear encoder and established that the real CTE is influenced by the bonding material between the aluminum extrusion and the glass scale. Moreover, during another study [39], a non-linear thermal behavior in optical linear encoders was noticed. This could be explained as a consequence of varying stresses transmitted from the extrusion to measuring scale.

The thermal error compensation could be an effective and economic method to improve optical encoder accuracy. This procedure is based on encoder error correction by introducing correction coefficients derived by using various mathematical ways and experimental research. Yu et al. [40] improve the rotary encoder accuracy by using Fourier expansion-polynomial fitting technique. In 2020, Jia et al. [41] proposed the compensation approach based on Fourier expansion-back propagation neural network technique optimized by a genetic algorithm. This group of scientists minimized rotary encoder error from 110.2 arc sec to 2.7 arc sec. Hu et al. [42] used the empirical mode decomposition and the linear least square fitting methods for a linear encoder error compensation at different temperatures. In general, there is not much information about the real thermal behavior of optical encoders and their accuracy under a real ambient condition. Even less information is published about a practical realization capability of the embedded error compensation solution.

In a previous work [43], the theoretical investigation of the linear encoder thermal behavior was done using the finite element method. The performed computer simulation analyzed the occurring thermal processes and introduced thermoelastic deformations, when a linear encoder is influenced by various heat sources and the changing temperature of the working environment. The results showed that the analyzed encoder demonstrated systematic behavior, which could be approximated by a simple parametric function. This could be used to compensate the final encoder position value, in order to improve its accuracy.

In this work, the real-time geometric and thermal error compensation approach is proposed. The article is based on theoretical and experimental research of the tested optical linear encoder and practical realization of the composed compensation algorithm. The presented method is optimized by experimentally estimating actual CTE of the linear encoder under test and could reduce the thermoelastic error to the accuracy range specified by the manufacturer. In Section 2, the error compensation background is discussed. Section 3 presents the setup used for the experimental investigation. Equipment used for the tested encoder accuracy measurement at different ambient temperatures and the composed subsequent electronics which realize the error compensation in real-time are specified. Obtained results and performed compensation algorithm optimization, based on a real CTE calculation, are described in Section 4. The discussion about the collected data and a short summary are written in Section 5. In Section 6, the main findings and conclusions are listed.

## 2. The Error Compensation Method for Linear Encoder

The accuracy of the encoder is one of the most significant parameters. This term describes the difference between the target position (real position value) and actual position—the encoder position reading. Mathematically, the position reading of the encoder Q could be expressed as:

(1) $$Q=Q_{\mathrm{real}}+\delta ,$$

where $Q_{\mathrm{real}}$—is a real linear position value, δ—is an error component. Therefore, the accuracy of the encoder is directly related to the size of a measurement error and is sometimes called the position error. In practice, the encoder accuracy measurement is a specific procedure that requires a well-calibrated equipment and a certain environmental condition. The calibrating encoder readings are compared with a reference device position indication, which are accepted as a real position value $Q_{\mathrm{real}}$. Usually, second, a highly accurate encoder or a laser interferometer is used as a reference.

According to ISO 5725-1, when the accuracy term is applied to sets of measurements of the same measurand, it involves a component of systematic error and a component of random error [44]. In this case, the term “trueness” is used to describe the closeness of the mean of a set of measurement results to the actual value and term “precision” is used to describe the closeness of agreement among a set of results. In practice, the trueness is accepted as the accuracy of the encoder and the precision is used to describe the repeatability or reproducibility of the device. In general, linear encoder error δ depends on the position q and the temperature T. Then, the mathematical model of the error is written as:

(2) Δ(q,T)=F(q,T)+ε,

where ΔF(q,T)—parametric function approximating a systematic error component, ε is the residual random component. In optical linear encoders, glass or stainless steel scales with a precise grating patterns are used for a linear position measurement. The manufacturing inaccuracies, like a varying duty cycle of the grating; any kind of scale deformations during the encoder assembly or mounting procedure; and a thermal expansion or contraction of the encoder due to ambient temperature or other temperature sources cause the systematic error component that could be measured, approximated by a parametric function and compensated. The error compensation K is equated to the systematic error component approximating function value with an opposite sign, i.e.,

(3) K=−F(q,T).

Other effects, like accidental mechanical vibrations or a shock, dust, metal chips or any other contaminants on the measuring scale surface, etc. represent the random error component and cannot be easily compensated.

Theoretically, the simplified mathematical error model for compensation could be expressed as:

(4) $$\Delta \left(q,T\right)=F_{g}\left(q\right)+F_{\mathrm{gr}}\left(q\right)+F_{\alpha }\left(\Delta T,q\right),$$

where the first member is $F_{g}\left(q\right)$—geometric error approximation function. The calibration process of the encoder is done at nominal temperature $T_{n}=20{\;}^{\circ }C$, in a special thermostable laboratory room, where ambient temperature varies only about $\pm 0.2{\;}^{\circ }C$. Tested encoder error values are plotted in graph according to the linear position values. Such a plot is called an encoder accuracy graph and is added to each manufactured encoder as a document to ensure the accuracy of the calibrated device. In this case, the compensation model assumes, that the ambient temperature stays constant and is equal to a nominal $T=T_{n}$. The calibrated encoder error plot is approximated by a single parametric function whose argument is the position value q.

(5) $$\Delta \left(q\right)=F_{g}\left(q\right)+\varepsilon_{g},$$

The second member $F_{\mathrm{gr}}\left(q\right)$—thermoelastic error approximation, when thermal gradient is steady and ambient temperature is stable. In real applications, there are various temperature sources around the measuring system. Those sources generate a relative temperature gradient along the encoder. That causes an unwanted deformation of the measuring scale. Estimation of the approximating thermal error function consists of the temperature gradient measurement by means of temperature sensor values in multiple points along the encoder, and calculation of the thermoelastic deformation of the measuring scale by the finite element method. The simulated total displacement values of the scale are approximated by a single parametric function, which is later used to estimate the thermal gradient error size in compensation process.

The third member $F_{a}\left(\Delta T,q\right)$—thermal error component—expresses a linear deformation of the measuring scale due to changing ambient temperature.

(6) $$F_{a}\left(\Delta T,q\right)=\alpha_{\mathrm{corrected}}\cdot \Delta T\cdot q,$$

where $\alpha_{\mathrm{corrected}}$—corrected coefficient of linear thermal expansion (CTE) of the measuring scale and ΔT—ambient temperature difference from the nominal temperature $\left(\Delta T=T_{n}-T\right)$.

The total error value calculated according the Equation (4) could be considered as a size for the real-time compensation. The determined compensation value at specific position should be relatively added or subtracted from the encoder position readings, in order to get the compensated position value.

## 3. Experimental Setup

**Figure 1:**
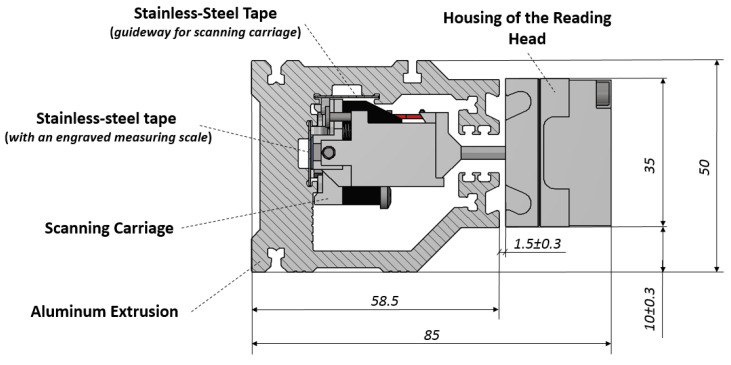
Cross-sectional view of the tested optical linear encoder LK50.

The prototype absolute linear optical encoder LK50 of the company JSC “Precizika Metrology” was chosen for the experimental research. It is a reflective type optical encoder with a measuring scale pattern engraved onto the stainless steel tape surface by a laser. The tape is fitted into the encoder’s aluminum extrusion, stretched by using a special rigid spring based mechanism and tightened at both ends. Another stainless steel tape is used as a guideway for a precise positioning and motion of the scanning carriage. The cross-sectional view of the tested optical linear encoder is shown in Figure 1.

The main parameters of the tested encoder, such as dimensions, measuring length and so on, are specified in Table 1.

In order to investigate the thermal behavior of the encoder, all experiments were carried out in a laboratory room, where the stable ambient temperature could be maintained. The specially customized technological stand was used for encoder mounting and imitation of an appropriate reading head motion along the measuring scale. The aluminum extrusion of the tested encoder was mounted onto the stainless steel support fixed on the granite base. The extrusion was attached with only one fixing screw in the middle of its length. In this way, the ends of the encoder could freely move during the thermal expansion and contraction. The reading head is attached to a moving carriage with an aerostatic bearing.

**Figure 2:**
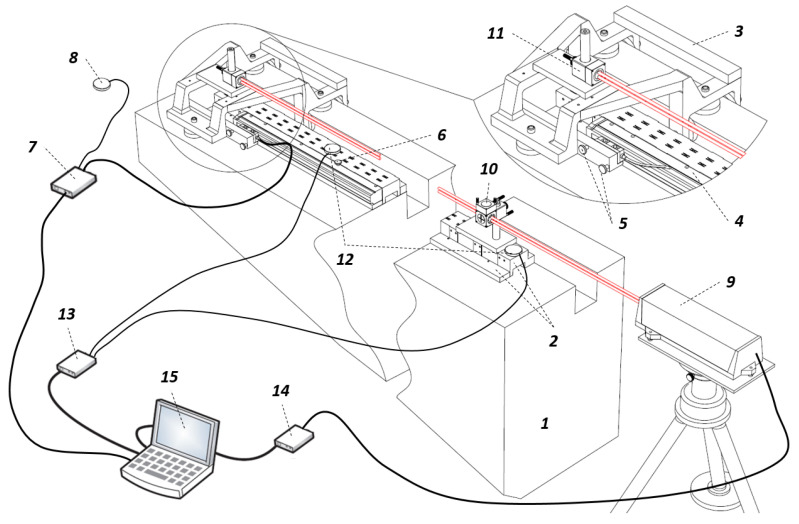
Experimental setup based on “Keysight 5530” system configured for a linear measurement.

During the tests, readings of the linear encoder were compared to a linear position indication of the laser calibration system “Keysight 5530”. The interferometer assembly was placed at the end of the technological stand. The retroreflector assembly is located at the moving carriage. To avoid uncertainties and compensate laser measuring system errors due to changing temperature, the “E1736A USB Sensor Hub” and relatively mounted temperature sensors “E1737A” were used. The composed experimental setup is shown in Figure 2.

The content of the used experimental setup is listed in Table 2 according the position numbers marked in Figure 2.

Considering the linear position compensation implementation into a real application, the response of the encoder becomes an important factor. For incremental encoders, the response is limited to a specific input signal frequency. The latency depends on the analog amplifier bandwidth, interpolation process, and the resolution. In practical applications, the incremental interface encoder latency is usually ignored, given that the edges of digital output signals have the real-time nature [45]. Unfortunately, the thermal error compensation process realization in incremental encoder is a hard task, because the output signals did not contain any information about the absolute position. They indicate the size of the reading head linear displacement. The absolute linear encoders usually consist of low resolution absolute position track and high resolution incremental track. The combination of the two tracks determines the absolute position value with a high resolution. These data are given on the demand of an application controller by a serial interface. The data transmission time depends on the bit length and overall speed.

The selected absolute optical linear encoder transmits its position by using a bidirectional synchronous serial interface BiSS. Usually it is used in industrial applications, where high transfer rates are required [46]. Depending on cable length, the encoder could handle clock frequencies up to 4 MHz and the calculation time is ≤5 µs. The maximum traversing speed is limited up to 2 m/s. The chosen resolution position is outputted with a 30-bit format. Taking into account the calculation time, the absolute position is transmitted upon ≤13 µs. Thermal and geometric encoder error compensation is realized by using the composed subsequent electronics. The programmable gate array (FPGA) platform “S7 Mini” with “Xilinx Spartan-7 7S25” is used as a master to request and get the linear encoder position readings, calculate the compensation value according to an integrated mathematical algorithm and external ambient temperature sensor data, and output the compensated position at the real-time. The vanishingly small calculation time of the FPGA could perform the compensation process almost instantly. If the compensation is processed by subsequent electronics, the FPGA has to receive, recognize, compensate, and generate the absolute position value. The whole process takes approximately double the time of the encoder transmission time. In this case, a compensated position is outputted ≤26 µs. If the proposed mathematical algorithm could be installed into the integrated FPGA or other controller, the calculation time might be drastically reduced.

The measuring length of the tested linear encoder is 1200 mm. The rectilinear velocity of the moving carriage is 0.2 m/s. To reduce the uncertainty and maintain the reproducibility of the successive measurements, the digital incremental encoder signals are also recorded. According the counted edges of these signals, the absolute position request is sent to the encoder at every 1000 counts i.e., at each 0.1 mm. In such a way, there are 12,000 equally spaced measured positions along the linear encoder. 

**Figure 3:**
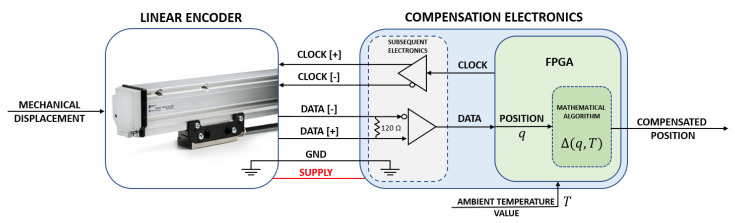
Block diagram of the compensation electronics with a field-programmable gate array (FPGA).

Additionally, the “Texas Instruments” THVD 1451 RS-485 transceivers are used to deal with differential encoder CLOCK and DATA signals. The simplified block diagram of the composed compensation electronics is shown in Figure 3.

The encoder readings, compensated encoder position values, and respective indications of the laser interferometer measuring system are recorded simultaneously during all experiments. Collected data are processed by using a numerical computing software environment “MATLAB” for the estimation of approximating function, further data analyzation, and graphical representation.

## 4. Results

Firstly, the whole experimental setup was left in the laboratory room at fixed nominal ambient temperature $T_{n}=20\pm 0.2{\;}^{\circ }C$ for 5 h to stabilize. During the tests, the ambient temperature was changed, so this stabilization process was repeated four times, at each settled temperature (i.e., $20{\;}^{\circ }C,\;17.8{\;}^{\circ }C,\;22.6{\;}^{\circ }C\;$, and $25.3{\;}^{\circ }C$). Because in the laboratory room there were a number of electronic components which generate approximately the same amount of heat all the time, it is stated that along the linear encoder existed a steady thermal gradient. Temperature differences in various part of the encoder induced thermoelastic deformations of the measuring scale. Encoder mounting could also be the source of the linear position measurement error, because of misalignment or deformations during fixation, lack of support stiffness, inaccurate guideway of the carriage, and so on. All these factors introduced the geometric error component. These conditions are relatively close to some of a real application, where such an encoder could be used.

Five separate unidirectional measurements are taken at each temperature. The average value of these five measurements is calculated. Based on standard ISO 230-2, the half peak-to-peak value of the resulting average position error curve is accepted as the unidirectional systematic positioning error of the encoder. The compensation and minimization of this systematic error is the main goal of this work.

**Figure 4:**
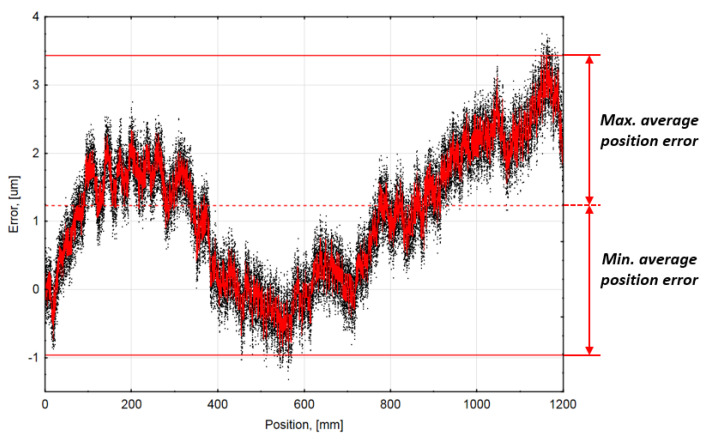
Accuracy graph at 20 °C ambient temperature. (Black dots—the measured error values at corresponding positions; Red curved line—the resulting average position error curve).

The first five measurements at 20 °C ambient temperature were recorded. The error values at corresponding positions and the average meaning curve are presented in Figure 4. The unidirectional systematic positioning error of the encoder was ±2.2 µm. This value is accepted as the accuracy of non-compensated tested encoder.

**Figure 5:**
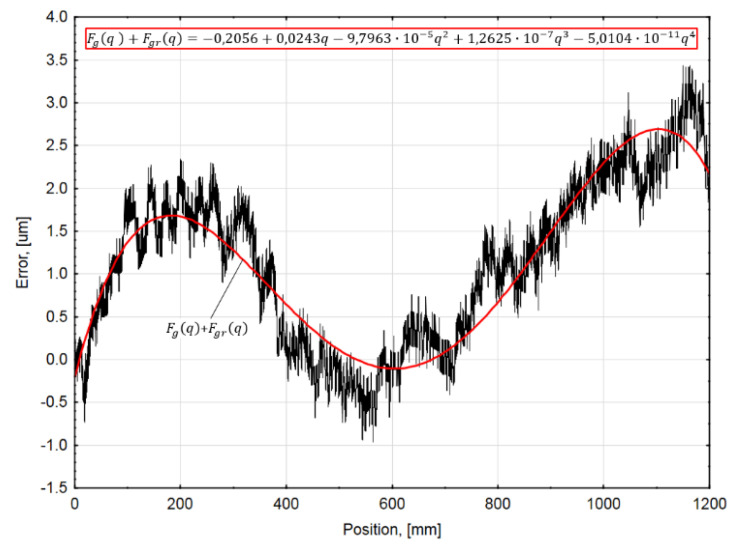
Average accuracy graph at 20 °C ambient temperature approximated by a parametric function. (Red line—approximating function and its equation).

The parametric function approximating the average position error curve could be accepted as the combination of the geometric $F_{g}\left(q\right)$ and the thermoelastic $F_{\mathrm{gr}}\left(q\right)$ error components, i.e., the sum of the first two members of the Equation (4). The fitted approximating function is shown in Figure 5.

The graph data are quite accurately approximated by a 4th order polynomial function that could be described by the following equation:

(7) $$F_{g}\left(q\right)+F_{\mathrm{gr}}\left(q\right)=F_{G}\left(q\right)=-0.2056+0.0243q-9.7963\times 10^{-5}q^{2}+1.2625\times 10^{-7}q^{3}-5.0104\times 10^{-11}q^{4},$$

**Figure 6:**
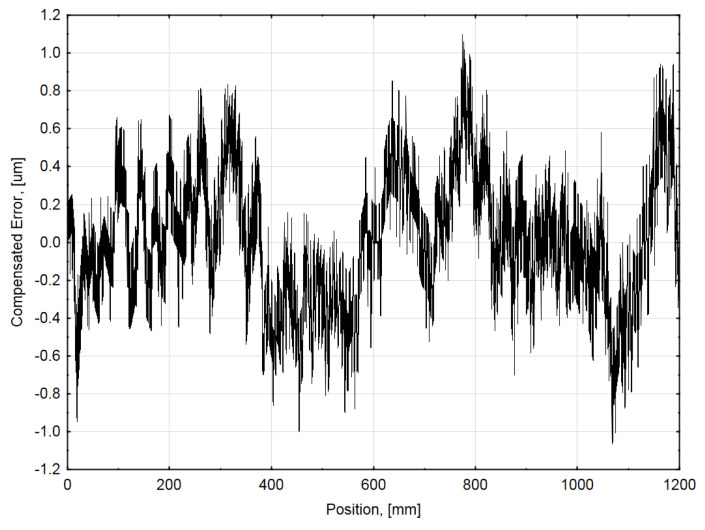
Average accuracy graph of compensated tested encoder at 20 °C ambient temperature.

This determined function was used as a base for the further thermal and geometric error compensation value calculation. The approximating function was integrated into compensation electronics (FPGA), and the five measurements were repeated. The accuracy graph of an average position error of the compensated linear encoder is shown in Figure 6.

**Figure 7:**
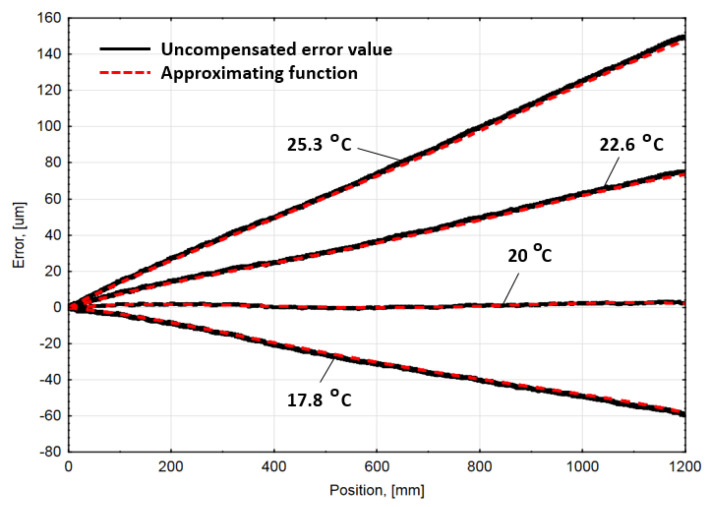
Combined average accuracy graph of uncompensated linear encoder average error at different ambient temperatures, and their compensation functions calculated according the presented mathematical algorithm.

In order to minimize encoder error at different ambient temperatures, the third component of the general error Equation (4) must be found. The mechanical construction and the thermal behavior of the linear encoder were investigated in a previous work [43] by using the finite element method (FEM). The computer simulation showed that the linear thermal expansion coefficient (CTE) of the measuring scale was greatly changed because of its fixing type, mass, and geometry differences between the scale and the extrusion, etc. Due to these reasons, the CTE of the scale was increased up to $\alpha_{\mathrm{corrected}}=22.9\;µm/\left({m\;}^{\circ }C\right)$. This value was used for the third member calculation, according the Equation (6). The ambient temperature difference from the nominal temperature was calculated according to the mean value of several external temperature sensor readings. The mathematical algorithm was supplemented and integrated into compensation electronics for other experiments at different ambient temperatures. The average uncompensated linear encoder accuracy graphs and the compensation functions, calculated according the mathematical algorithm, are combined in Figure 7.

How accurately the derived function describes the average uncompensated encoder error was evaluated by a standard deviation of error meanings with respect to the determined function.

(8) $$\sigma_{G}=\sqrt{\frac{\sum_{i=0}^{N}{\left(\bar{x_{i}}-F_{G}\left(q_{i}\right)\right)}^{2}}{N}},$$

where $\sigma_{G}$—a standard deviation, $\left(\bar{x_{1}},\bar{x_{2}},\ldots ,\bar{x_{N}}\right)$—values of calibration process realizations, $q_{i}$—value of the argument q (linear position), which help to estimate the compensation function value $F_{G}$, and N—number of realizations. To evaluate ~96% of measurements, the standard deviation was multiplied by 2.1. Both values for each ambient temperature are listed in Table 3.

**Figure 8:**
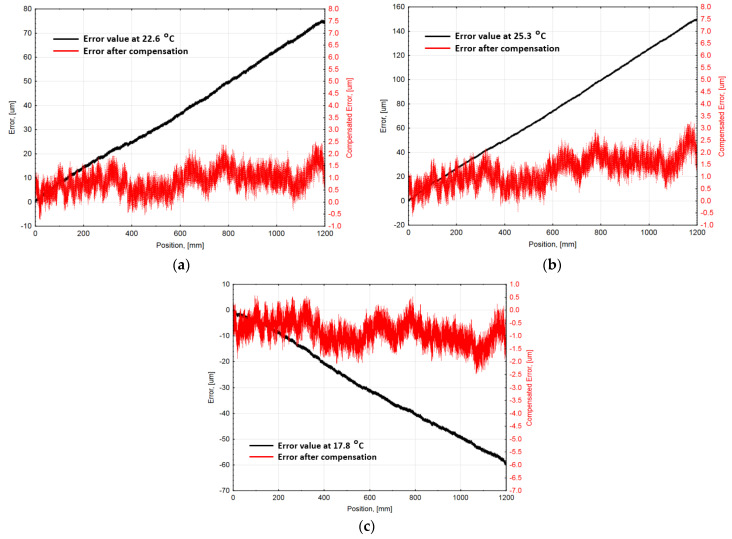
Compensated linear encoder average accuracy graphs (red line/right Y-axis units) with corresponding uncompensated error values (black line/left Y-axis units), at different ambient temperatures: (**a**) at 22.6 °C; (**b**) at 25.3 °C; and (**c**) at 17.8 °C.

After applying the specified functions, the compensation electronics gave corrected position values. The average compensated linear encoder accuracy graphs and corresponding average uncompensated error values are shown in Figure 8.

The main indicators, such as maximum and minimum values and unidirectional systematic encoder error (average encoder accuracy), etc., are listed in Table 3.

### 4.1. Estimation of the Real Thermal Coefficient

The accuracy of the compensating value calculation highly depends on how precisely the approximating function is fitted to measured data. Unfortunately, the higher order polynomials or even more complex interpolation functions could cause a practical problem with their integration and calculation time. More efficient and more expensive calculation platforms could be needed. Another way to improve the precision of the presented approach is to estimate the real coefficient of the linear thermal expansion (CTE). Because the mounting of the tested encoder during the performed experiments allows the free axial movement (the encoder could freely expand and contract), the real CTE estimation could be done by analyzing the experimental data of average uncompensated encoder error values.

**Figure 9:**
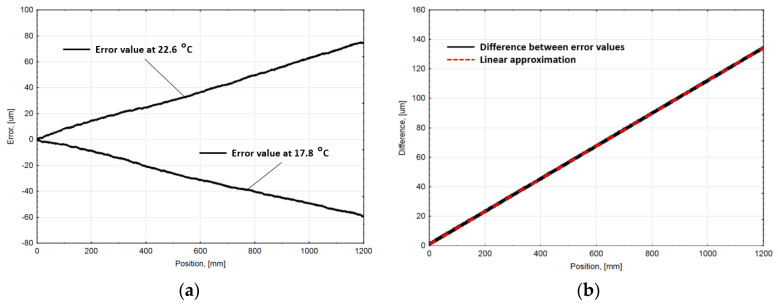
(**a**) Accuracy graphs of uncompensated linear encoder at $17.8{\;}^{\circ }C$ and $22.6{\;}^{\circ }C$; (**b**) Differences between accuracy graphs and linear fitting line to the difference.

The average accuracy graphs obtained at stable $17.8{\;}^{\circ }C$ and $22.6{\;}^{\circ }C$ ambient temperatures were taken. For a detailed interpretation, both graphs are represented in Figure 9a. The difference between the values of the graphs was calculated at every particular position and plotted in Figure 9b. The received meanings of the differences were approximated with the linear regression line, whose equation is:

(9) y=0.1111q+1.011,

where y—approximated value of the differences, q—is the position value, number 0.1111 is a constant that represents the slope of the linear regression line, and constant 1.011 is the ordinate at the origin.

The real CTE $\alpha_{\mathrm{Real}}$ is estimated as the ratio between the determined approximating line slope and the span of ambient temperatures [38]:

(10) $$\alpha_{\mathrm{Real}}=\frac{0.1111}{22.6-17.8}=23.15\;\left[\frac{µm}{m\cdot \circ C}\right],$$

The calculated value of the CTE is slightly different compared to the simulated $\alpha_{\mathrm{corrected}}$. This determined value is greater than the theoretical thermal coefficient, which was used in FEM simulation as aluminum extrusion material property ($23\times 10^{-6}\;m/\left(m^{\circ }C\right)$. This suggests that the real CTE of aluminum extrusion is greater. In the literature, the CTE of the aluminum varies from $23\times 10^{-6}m/\left(m^{\circ }C\right)$ to $24\times 10^{-6}m/\left(m^{\circ }C\right)$. Such an experimental result allows to improve the computer model and the precision of the presented compensation algorithm.

### 4.2. Recalculation of the Compensated Results According to the Real CTE

**Figure 10:**
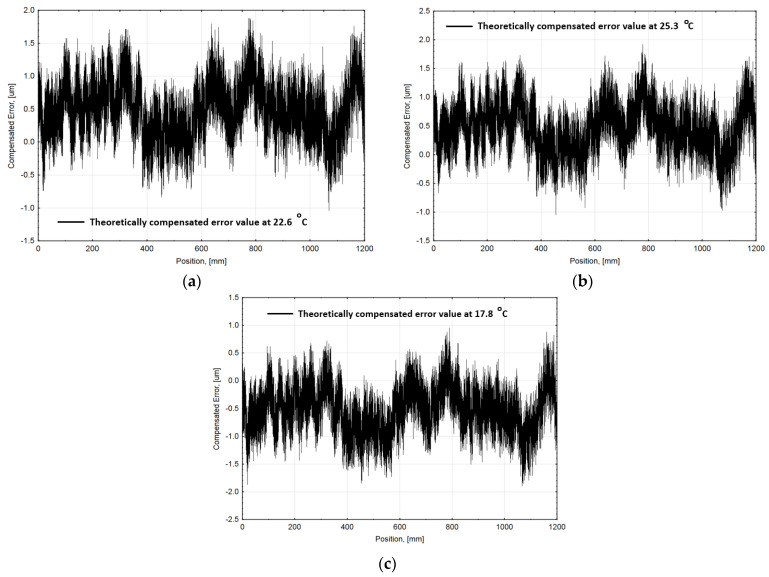
Theoretically recalculated compensated linear encoder accuracy graphs at different ambient temperatures: (**a**) at 22.6 °C; (**b**) at 25.3 °C; and (**c**) at 17.8 °C.

The presented compensation algorithm was adjusted by including the estimated real thermal coefficient into the Equation (6). The compensated error values at different ambient temperatures were calculated and subtracted from the average uncompensated encoder values, recorded during the experiments. This allowed to determine the influence of the introduced changes and compare it to the performed test data. The recalculated compensated error graphs are shown in Figure 10.

The accuracy of approximating compensation functions based on a standard deviation and the parameters of compensated encoder errors are listed in Table 4.

## 5. Discussion

The proposed mathematical algorithm is based on the approximation of geometric and thermoelastic linear encoder error by a simple parametric function and calculation of linear nature thermal error component in accordance to varying ambient temperature. Based on previous work’s [46] accomplished computer modeling results and the practical tests, such a compensation technique was adapted to the selected linear optical encoder.

Performed experiments demonstrated that the designed subsequent electronics are suitable for the realization of the presented compensation approach. An FPGA-based calculation platform properly read tested linear optical encoder position value and compensated it according the installed mathematical function. All processes were performed in real-time; therefore, it could be applied into an industrial, scientific, or other technological application.

The experimental results demonstrate that geometric and introduced thermoelastic linear position measurement error could be drastically reduced:

- At nominal $20{\;}^{\circ }C$ ambient temperature, measured encoder average accuracy was ±2.20 µm. After the average position error graph approximation and position compensation, the recorded error was minimized to ±1.08 µm. The approximation accuracy evaluating ~96% measured positions reached ±0.72 µm.
- At different ambient temperatures ($17.8{\;}^{\circ }C$; $22.6{\;}^{\circ }C$ and $25.3{\;}^{\circ }C)$, encoder average accuracy respectively reached ±30.08 µm; ±37.74 µm, and ±75.09 µm without compensation. Applied mathematical algorithm at these temperatures could approximate encoder error correspondingly: ±2.06 µm; ±2.14 µm, and ±2.94 µm. Considering that the maximal error value reaches up to ~150 µm, the average accuracy of approximation was accepted as reasonable.
- After the position compensation process, the average encoder accuracy at different temperatures was determined as the following: ±1.52 µm (at $17.8{\;}^{\circ }C$); ±1.62 µm (at $22.6{\;}^{\circ }C$), and ±1.95 µm (at $25.3{\;}^{\circ }C$). Considering that the specified accuracy of a standard encoder is ±5 µm per meter, the compensated average encoder accuracy (including the uncertainty of the approximation at different temperatures) was within this range. It can be stated that the performance of the encoder remained under different thermal environmental conditions.
- The presented algorithm could be optimized according to experimentally estimated real CTE value. Embedding this value into the compensation allowed to improve the accuracy of the encoder error approximation which in turn decreased the total error. Theoretical calculations show that the encoder accuracy could reach: ±1.42 µm (at $17.8{\;}^{\circ }C$); ±1.46 µm (at $22.6{\;}^{\circ }C$); and ±1.48 µm (at $25.3{\;}^{\circ }C$).

## 6. Conclusions

The approach of a thermal and geometric error compensation for a linear encoder is introduced in this article. Having designed the suitable technological equipment, performed experimental research, and analyzed the systematized results, the following conclusions are drawn:

1. The thermoelastic linear encoder deformation caused by external heat sources and changing ambient temperature is significant. Considering the linear thermal expansion coefficient, which greatly depends on an encoder design and used materials, and the working environment conditions, the linear position measurement uncertainty could have a big numerical value. This could lead to undesirable performance of the encoder or even a whole application.
2. The proposed error compensation model is suitable for thermoelastic and geometric error compensation. The performed experiments show that the introduced tested encoder error could be significantly reduced up to 98%. Usage of this kind’s compensation might be cheaper and more appropriate solution compared to others, like encoder design including close to zero thermal expansion materials or control of working environment temperature.
3. The compensation algorithm implementation into FPGA-based calculation platform demonstrates the reliable performance. Such hardware selection can ensure an appropriate calculation speed for a real-time application. Due to its flexibility and low cost, it is possible to integrate this device into encoder design or use it like a subsequent electronics module.

However, certain details still exist that require in depth theoretical and experimental research, such as the dynamically changing temperature gradients, different encoder designs, and mounting methods, etc.

## Figures and Tables

**Table 1:** Parameters of tested optical linear encoder.

Concept	Value	Units
Measuring length (ML)	1200	mm
Accuracy (to any meter within the ML)	±5	µm/m
Resolution	0.1	µm
Interface	BiSS-C	-
Aluminum extrusion	Dimensions: 50 × 58.5 × 1485	mm × mm × mm
	Thermal coefficient (CTE): 23 × 10^−6^	m/(m °C)
Stainless steel tape	Dimensions: 12 × 0.5 × 1440	mm × mm × mm
	Thermal coefficient (CTE): 10.5 × 10^−6^	m/(m °C)

**Table 2:** Content of the experimental setup.

Position	Object
1	Granite base
2	Stainless steel support (for encoder mounting)
3	Moving carriage (with aerostatic bearings)
4	Optical linear encoder (device under test)
5	Fixing screws (for encoder reading head)
6	Fixing screw (for encoder aluminum extrusion)
7	Subsequent electronics (for error compensation)
8	Ambient temperature sensor (E1738A)
9	Laser (5519A/B)
10	Interferometer assembly (linear interferometer, linear retroreflector, base, height adjuster, and post)
11	Retroreflector assembly (linear retroreflector, post and height adjuster, base)
12	Temperature sensors (E1737A)
13	USB sensor hub (E1736A)
14	USB axis module (E1735A)
15	PC (with an appropriate software)

**Table 3:** Parameters of approximating function and compensated tested encoder accuracy graphs.

Parameter	Ambient Temperature			
	17.8 °C	20 °C	22.6 °C	25.3 °C
Accuracy of approximating function (by mean of standard deviation)	±0.98 µm	±0.34 µm	±1.02 µm	±1.40 µm
Std. dev. of ~96% measurements (Std. dev. multiplied by 2.1)	±2.06 µm	±0.72 µm	±2.14 µm	±2.94 µm
Maximal error value (Non-compensated encoder)	0.07 µm	3.43 µm	75.47 µm	150.03 µm
Minimal error value (Non-compensated encoder)	−60.09 µm	−0.96 µm	0.01 µm	0.15 µm
Average accuracy of non-compensated encoder	±30.08 µm	±2.20 µm	±37.74 µm	±75.09 µm
Maximal error value (Compensated encoder)	0.57 µm	1.10 µm	2.51 µm	3.26 µm
Minimal error value (Compensated encoder)	−2.48 µm	−1.07 µm	−0.73 µm	0.64 µm
Average accuracy of compensated encoder	±1.52 µm	±1.08 µm	±1.62 µm	±1.95 µm

**Table 4:** Parameters of approximating function and theoretically calculated encoder accuracy graphs (including experimentally estimated real thermal coefficient).

Parameter	Ambient Temperature		
	17.8 °C	22.6 °C	25.3 °C
Accuracy of approximating function (by mean of standard deviation)	±0.69 µm	±0.67 µm	±0.65 µm
Std. dev. of ~96% measurements (Std. dev. multiplied by 2.1)	±1.45 µm	±1.41 µm	±1.35 µm
Maximal error value (Compensated encoder)	0.95 µm	1.88 µm	1.92 µm
Minimal error value (Compensated encoder)	−1.89 µm	−1.04 µm	−1.04 µm
Compensated encoder accuracy	±1.42 µm	±1.46 µm	±1.48 µm

## Data Availability

Not applicable.

## Abbreviations

The following abbreviations are used in this manuscript:

CTE	Coefficient of Thermal Expansion
FPGA	Field-programmable Gate Array
